# What They Think of Us

**Published:** 1858-06

**Authors:** 


### WHAT THEY THINK OF US.

Dr. Campbell Stewart having made some written statements of the condition of "Medicine in the United States," a French Journal remarks as follows:

In the United States, medicine is in no way under the protection or supervision of the Federal Government. Each State individually is charged with the care, of which it relieves itself by conferring on the universities or colleges the power of granting diplomas. There are in the Union at least forty universities or colleges in which medicine is regularly taught.

The organization of the schools, the regulations in force, and the subjects taught do not differ from what they are with us, as much as one might suppose. The regulation of the University of Pennsylvania shows that as far as medicine is concerned, the principal difference consists in the greater facility allowed to students for acquiring their degrees. To be twenty-one years of age, three years a student, during which he shall have attended two full courses of lectures at the university, and moreover to be a private student of a commendable practitioner; such are the only conditions required of the candidate for the degree of doctor. A single examination confers this title upon him. The day having come, the candidate addresses a petition to the dean, in sending him a thesis upon some medical subject of his own choice. The thesis is laid before one of the professors, who examines it, interrogates the candidate, and transmits his notes to the faculty, who decide of his nomination by secret ballotting. And besides, the examination does not take place in a uniform manner in all the schools. Sometimes it is public; sometimes it takes place before the professors and the trustees of the school alone; then again, as at the University of New York for example, there is properly speaking no examination at all; each of the professors produces his notes on the candidate; the faculty then assembles, and when the dean calls the name of the candidate, the faculty consults the notes received and vote thereby. If there are three votes against the candidate, or even two in some of the schools, he is rejected and required to attend another session.

The number of professorships in each school is from five to nine, more generally seven or eight. General and descriptive anatomy, the history of medicine, pathology, the materia medica, therapeutics and pharmacy, chemistry and toxicology, obstetrics, the diseases of women and children, legal medicine: such are the subjects taught. The course commences in October, and generally terminates in March, the epoch at which the students undergo their examination. The time that they devote to the course is from five to six hours per day, besides the time occupied in dissections and visits to the hospitals.

It will be perceived from these details, that the degree of medical instruction in the United States is little elevated relatively to what it is with us. The time devoted to study is shorter, the subjects taught are less varied, the courses are fewer in number and more superficial, and the acquirements of the student are guaranteed by only one physician. But if we will reflect on the great increase of the population in the United States, the incessant demand for physicians in every part of the country, and the nature of the institutions which leaves the door open to the most shameless charlatanism, we will comprehend that it would be difficult for it to be otherwise, and that a great number of obstacles thrown in the way of medical instruction would result in turning a certain number of young men away from serious studies, although incomplete, which they prosecute in the schools.

However, medical instruction in America possesses one incontestible advantage over ours, in the kind of apprenticeship that each student serves under a learned and commendable physician, who initiates him into the difficulties of practice. Each student thus attaches himself to a practitioner, under whose observation he is constantly placed; he follows him to his consultations, accompanies him in his visits, and assists him in his operations. Sometimes the physician even lodges the student in his own house, makes him labor under his own eyes, and in frequent conversations endeavors to direct his mind towards reflection and study, and to develop the aptitudes that he discovers in him.

Neither are the elements of instruction wanting in the United States. They have hospitals, amphitheatres, museums of pathological anatomy, laboratories, libraries, and medical presses. In the city of New York and its environs, there are three great hospitals, each of which accommodates more than six hundred patients, and still they are building a fourth; two clinical establishments for those laboring under diseases of the eyes and ears, four dispensaries, two asylums for the insane, two lying-in hospitals; a hospital for the diseases of females, the quarantine hospital for the treatment of contagious and infectious diseases; a hospital for the sailors of the merchant marine, and another for those of the state, and finally another for the reception of sick children. The various establishments are all open to the students at little or no expense, and these facilities given to clinical studies have given them a very great repute.

It has only been a short time that as much could be said of dissections; subjects could only be obtained clandestinely. It appears that public opinion is now so much modified upon this subject, as the government of New York has ceded to the just demands of physicians, by encouraging the study of pathological anatomy, (which will hereafter be done publicly), and enabled scientific interest to triumph over ignorance and prejudice.

After this rapid sketch of medical teaching in America, let us pass on to the practice; and let us consider without prejudice the situation of the physicians in the United States. The law accords to him no protection whatever; in some States protective laws do not exist at all, in others they exist but in the state of a dead letter, and no one thinks of claiming their application. The field is open, and all are free to enter it. Public opinion would regard with a suspicious eye every effort having for its object the regulation of such a license. A fact which forcibly impressed us was to see Doctor Campbell accept so deplorable a state of things with a certain satisfaction. He did not deny that he had formerly thought differently of the interference of government in medical affairs; but, better informed now, he thinks the good sense of the people will protect them against the encroachments of charlatanism better than all the laws that could be enacted on the subject. In short, he is of the opinion that things are well as they are, and that it would be wrong to change them in any way whatever.

It is true that Doctor Campbell informs us that the honorable and enlightened physician occupies an enviable position in America—often a brilliant one; that he enjoys a consideration and an influence that the members of the clergy themselves are scarcely able to equal; that he is often called to the highest political stations, and that the Senate and Legislative chambers always reckon members of the profession among their numbers.

To think only of this seducing picture, we would be willingly disposed to share the optimism of Doctor Campbell, and to demand the *statu quo*; but the colors with which, a little farther on, he paints medical charlatanism, are of a nature to somewhat moderate our joy.

However this may be, the consumption curers, who are a variety of the species, are not wanting in a certain originality—in evidence of which it will be sufficient to read the lamentable and amusing recital of a poor Virginia doctor, in the third stage of consumption, whose misfortune conducted him into the hands of these charlatans. One would imagine himself in the midst of an industrial establishment. The patient traverses a series of saloons full of employees: one precusses him, another auscults him. Every one has his speciality; this one examines the heart, that one verifies the condition of the lungs, and another (this is the most important personage) makes the prescriptions. This would be pleasant if it were less sad.

How then shall we appreciate the position of American physicians besides these patented quacks, warmly supported, forming a semi-official corps, who annually spend large sums in circular advertisements? We know that in the United States the industrial question over-rides every other consideration, and that the spirit and character of the American people accommodate themselves without scruple to all that would yet shock us; let us at least felicitate ourselves that we have not yet entirely reached such a point in France.—*Journal of Medicine and Surgery, Nashville.*

